# Effect of *Cannabis sativa* L. extracts, phytocannabinoids and their acetylated derivates on the SHSY-5Y neuroblastoma cells’ viability and caspases 3/7 activation

**DOI:** 10.1186/s40659-024-00506-0

**Published:** 2024-05-27

**Authors:** Elizabeth Tapia-Tapia, Pablo Aránguiz, Rodrigo Diaz, Luis Espinoza, Caroline R Weinstein-Oppenheimer, Mauricio Cuellar

**Affiliations:** 1 Escuela de Química y Farmacia, Facultad de Farmacia, Universidad de Valparaíso, Av. Gran Bretaña 1093, Playa Ancha, 2360102 Valparaíso, Chile; 2 Escuela de Química y Farmacia, Facultad de Medicina, Universidad Andrés Bello, 2520000 Viña del Mar, Chile; 3 Escuela de Ciencias de la Salud. Carrera de Química y Farmacia, Universidad Viña del Mar, 2572007 Viña del Mar, Chile; 4 Centro de Micro-Bioinnovación (CMBi), Universidad de Valparaíso, Av. Gran Bretaña 1093, Playa Ancha, 2360102 Valparaíso, Chile; 5 Departamento de Química, Universidad Técnica Federico Santa María, Avenida España 1680, 2340000 Valparaíso, Chile; 6 Centro de Investigación, Desarrollo e Innovación de Productos Bioactivos (CInBIO), Universidad de Valparaíso, 2360102 Valparaíso, Chile

**Keywords:** Cannabinoids, Cannabinol, Caspases, Cell viability, Δ9-tetrahidrocannabinol, Tetrahidrocannabinolic acid

## Abstract

**Background:**

There is a need for novel treatments for neuroblastoma, despite the emergence of new biological and immune treatments, since refractory pediatric neuroblastoma is still a medical challenge. Phyto cannabinoids and their hemisynthetic derivatives have shown evidence supporting their anticancer potential. The aim of this research was to examine Phytocannabinoids or hemisynthetic cannabinoids, which reduce the SHSY-5Y, neuroblastoma cell line’s viability.

**Methods:**

Hexane and acetyl acetate extracts were produced starting with Cannabis sativa L. as raw material, then, 9-tetrahidrocannabinol, its acid counterpart and CBN were isolated. In addition, acetylated derivatives of THC and CBN were synthesized. The identification and purity of the chemicals was determined by High Performance Liquid Chromatography and ^1^H y ^13^C Magnetic Nuclear Resonance. Then, the capacity to affect the viability of SHSY-5Y, a neuroblastoma cell line, was examined using the resazurin method. Finally, to gain insight into the mechanism of action of the extracts, phytocannabinoids and acetylated derivatives on the examined cells, a caspase 3/7 determination was performed on cells exposed to these compounds.

**Results:**

The structure and purity of the isolated compounds was demonstrated. The extracts, the phytocannabinoids and their acetylated counterparts inhibited the viability of the SHSY 5Y cells, being CBN the most potent of all the tested molecules with an inhibitory concentration of 50 percent of 9.5 µM.

**Conclusion:**

Each of the evaluated molecules exhibited the capacity to activate caspases 3/7, indicating that at least in part, the cytotoxicity of the tested phytocannabinoids and their hemi-synthetic derivatives is mediated by apoptosis.

**Supplementary Information:**

The online version contains supplementary material available at 10.1186/s40659-024-00506-0.

## Background

**Fig. 1:**
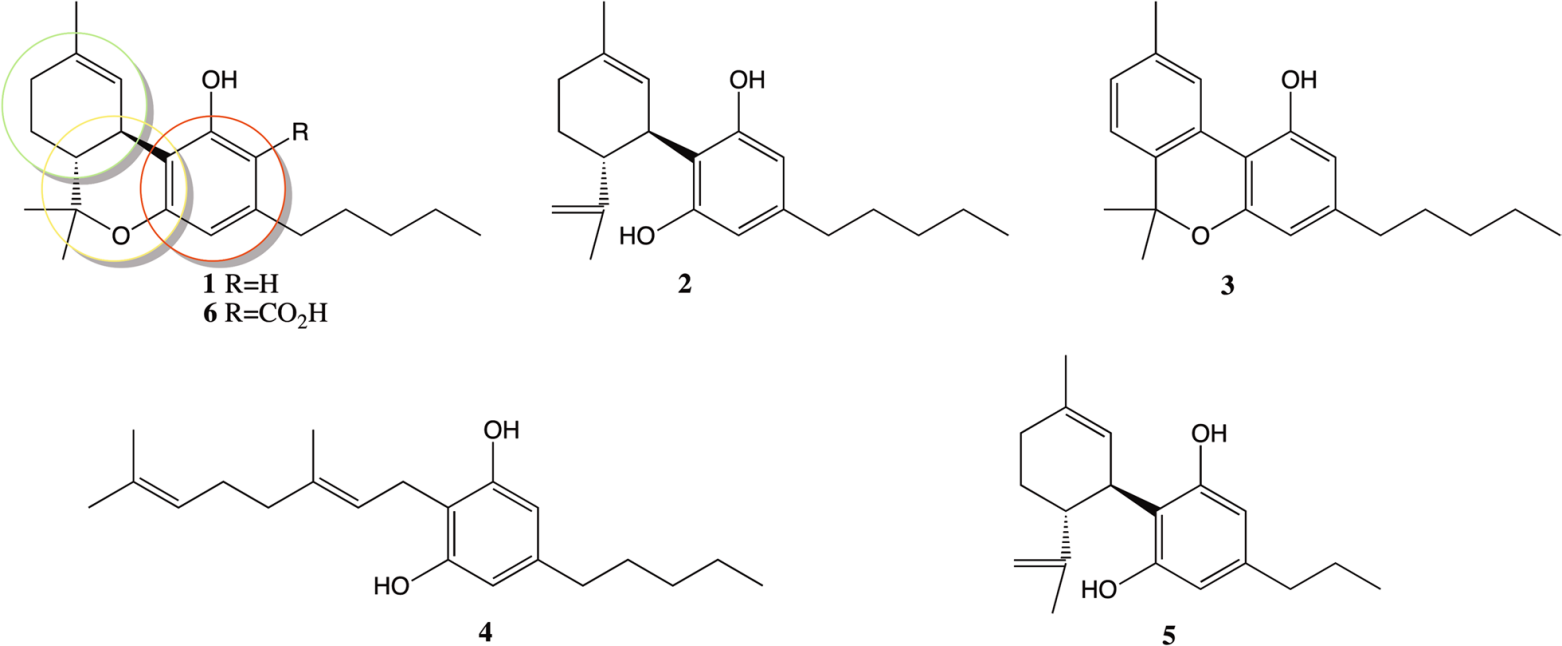
Main natural cannabinoids. The structures of Δ9-tetrahydrocanabinol (**1**), cannabidiol (**2**), cannabinol (**3**), cannabigerol (**4**), cannavidavarin (**5**) and tetrahydrocanabinolic acid (**6**) are shown. The green ring points at the ciclohexen ring, the yellow ring points to the tetrahydropyran ring, and the red ring corresponds to the benzene

*Cannabis Sativa* L. is an herbaceous plant, belonging to the *Cannabaceae* family [1]. One of the first studies aimed to the isolation and identification of cannabinoids was performed by Gaoni and Mechoulam. They isolated 9-tetrahidrocanabinol (THC), cannabinol CBN, cannabidiol (CBD) and cannabigerol (CBG) [2]. These compounds are generally formed by 21 carbons and have three rings in their structure, one ciclohexen (shown in green in Fig. 1) one tetrahydropyran (shown in yellow in Fig. 1) and one benzene (shown in red in Fig. 1).

Δ9-tetrahidrocannabinol (THC) is the most potent and abundant psychostimulant in the plant, cannabidiol (CBD) is another important metabolite that shows no psychoactive properties, and it is known by its anti-epileptic, antioxidant, and analgesic effects [3]. On the other hand, cannabinol (CBN) is not produced by the plant's metabolism, but it is a by-product of THC during the drying, storage and/or consumption of cannabis products [4].

It has been shown that cannabinoids affect signaling pathways associated with cancer, such as the ERK, GSK-3 alpha/beta and AKT pathways [5]*.* Physiological cannabinoids, called endocannabinoids, as well as phytocannabinoids and their hemisynthetic derivatives exert their effects mainly through specific receptors called CB1 and/or CB2. These are G*i/*o-coupled receptors whose main effectors are adenylate cyclase and potassium channels. CB1 is primary associated with neurotransmission and CB2 with functions associated to the immune system, including inflammation, cell migration and cytokines release [6]. The latter and the signaling pathways triggered by the mentioned receptors might give a rational for the observations made by several authors showing antiproliferative and antitumoral actions for cannabinoids.

One of the first investigations about the potential of cannabinoids as anticancer therapy was in 1975 when Munson et al. using an in vivo murine model showed a reduction at the size of a Lewis lung adenocarcinoma with oral therapy of THC. On the contrary, CBD showed no antitumoral activity [7]. After that, several studies have been performed aiming to search for a promising and very much needed new anticancer drug. Thus, the antiproliferative activity of phyto cannabinoids that show affinity for CB´s receptor, such as THC and CBN, the ones that do not bind to these receptors like CBD and CBG and synthetic cannabinoids with affinity for CB’s receptors were assessed on the highly aggressive MDA-MB231 breast cancer cell line. THC and CBN exhibited the highest potency for the inhibition of the breast cancer cell viability [8]. CBN’s in vitro inhibitory effect on human glioma, hepatocellular carcinoma and breast cancer cell lines' viability, that express high levels of the CB2 cannabinoid receptor, has been recently reported. Moreover, apoptosis has been suggested as one of the mechanisms of this effect [9]. THC and CBN have also been proven on the cholangiocarcinoma cell line HuCCT1, showing to inhibit cell proliferation at high concentrations and induce apoptosis which correlated with PARP cleavage. In addition, in that research an in vivo assay was conducted with tumor xenografted in nude mice, in which cannabinoids were injected daily around the tumors. It was shown that only THC exerted inhibitory effect over the tumor growth in vivo [5]. Interestingly, neuroblastoma cell lines have also been approached to examine the potential anticancer effects of cannabinoids, thus IMR-5 and SK-N-AS cells were shown to be inhibited on their viability and underwent apoptosis after being exposed to CBN [9]. Additional evidence was provided by studies with non-differentiated SH-SY5Y neuroblastoma cells showing that CBD increases cell death [10].

Neuroblastoma is an heterogenous kind of cancer, whose outcome may range from spontaneous regression to refractory disease. The later are, despite novel biological and immune treatments, still a challenge for pediatric oncologists [11]. With the evidence of phytocanabinoids, affecting neuroblastoma cells lines, we decided to examine Cannabis extracts, isolated Phyto cannabinoids and their hemisynthetic derivates on the non-differentiated SHSY5Y neuroblastoma cells to gain knowledge about its effect on cell viability and caspases 3/7 activation, as a marker of apoptosis induction. Besides, due to the promising properties of phytocannabinoids, researchers have focused on the synthesis of either the natural metabolites and/or their synthetic derivatives with the aim to improve efficacy and selectivity. For this reason, in this research, we also tested acetylated derivates of THC and CBN. To review the cytotoxicity of cannabinoids on a neuroblastoma cell line has two relevant purposes, on one hand to gain insight into the neurodegenerative potential that could be associated with the recreative effects of cannabis consumption, and by the other hand, to explore their potential pharmacological use as novel anti neoplasia agents. Thus, the search for compounds with comparable activity to THC but lacking its psychotropic effects offers an avenue in the search of the urgently needed novel anticancer drugs.

## Results

### Extraction and purification of cannabinoids

The *Cannabis sativa* L. vegetal material was used to produce two extracts using hexane and acetyl acetate as solvents. This allowed to have a starting material to obtain THC, CBN and THCA, the acid form of Δ9-tetrahidrocannabinol. CBN was not detected in the hexane extract and none of the extracts allowed to purify CBD. On Table 1 the yields of cannabinoids from the two extracts are shown. Hexane was the best solvent to isolate THC-A, whereas acetyl acetate was a better solvent to recover higher yields of THC and CBN.

**Table 1:** Yield of cannabinoids for different starting extracts

Compound	Yield (%)	
	Hexane	Acetyl acetate
THC	4.1	7.8
CBN	Not detected	3.3
THC-A	4.6	1.3

### Characterization of extracts, phytocannabinoids and their acetylated counterparts

#### High Performance Liquid Chromatography (HPLC)

**Fig. 2:**
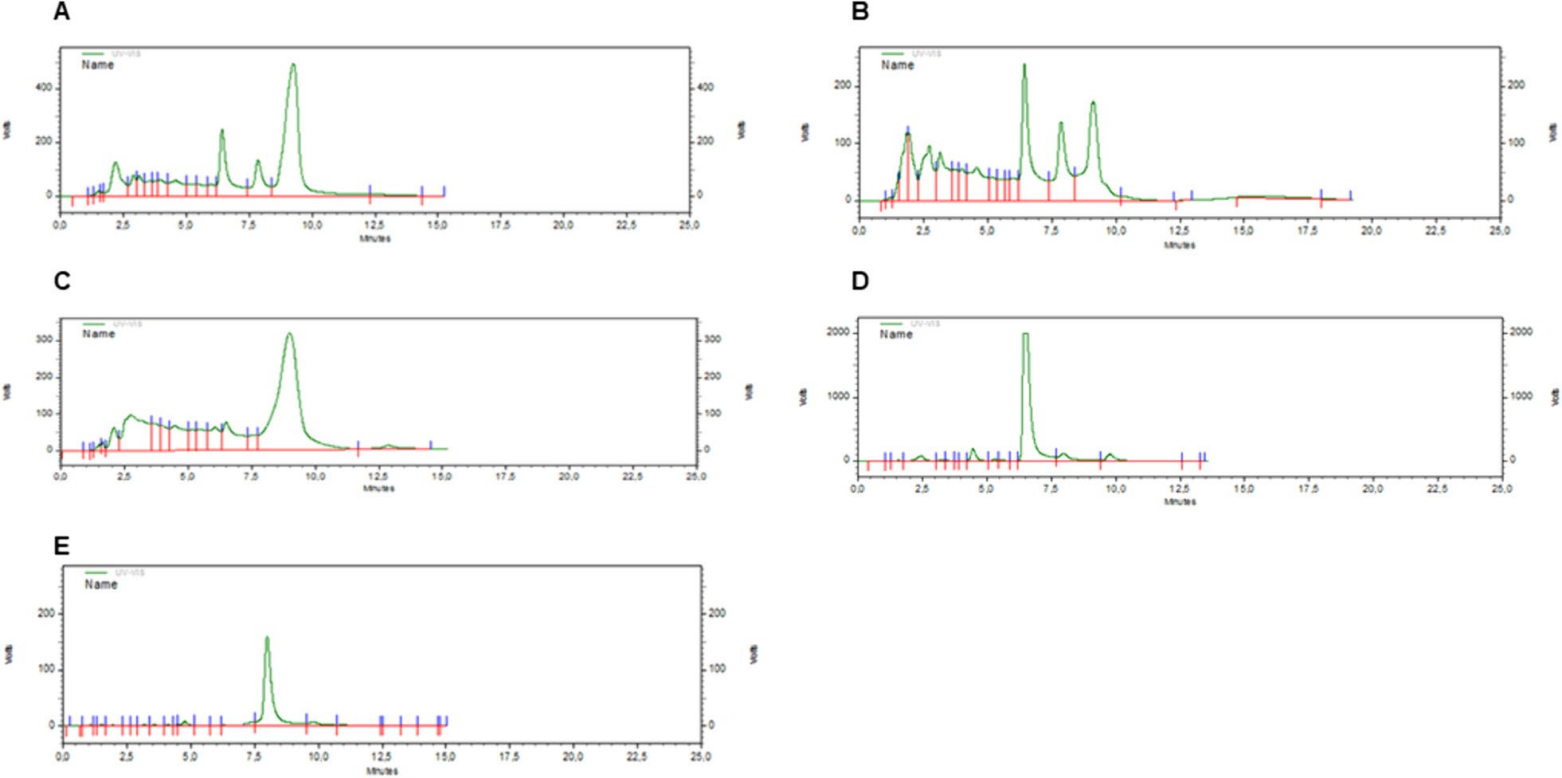
HPLC chromatograms of extracts and isolated Phytocannabinoids. **A** is the hexane extract, **B** is the acetyl acetate extract, **C** is THC, **D** is CBN and Panel E is THCA. The analysis was performed with a 20 μL sample injection in a Kromasil 100–5-C8 (4.6 × 150 mm) column with a water:acetonitrile (20:80) mobile phase at 1.0 mL/min. The detection was performed with an UV detector at 272 nm [12]

Extracts and Phytocannabinoid were analyzed by HPLC to gain insight on their degree of purification. The chromatograms are shown in Fig. 2. As it can be seen, both extracts showed their complexity as expected for a complex mixture of molecules. Conversely, THC appears as the main peak in the chromatograms with some degree of non-identified compounds. In addition, the CBN and THCA chromatograms showed that they were the main molecules present in the purified products.

#### Magnetic Nuclear Resonance (MNR)

The molecules were identified through their ^1^H and ^13^C RMN spectrum which were consistent with the expected chemical structure for THC, THCA, THC-Ac, CBN and CBN-Ac and previously reported assignations [13]. The signals for each molecule are presented below and their spectrum can be observed in supplementary Figs. 2–10.

##### 9-tetrahidrocannabinol (THC)

^1^H RMN (400 Mz, CDCl_3_, δ, ppm): 6.34 (1H, sa, H-2); 6.28 (1H, s, H-5ʹ); 6.15 (1H, d, *J* = 1.0 Hz, H-3ʹ); 5.18 (1H, sa, 2ʹ-OH); 3.22 (1H, d, *J* = 10.9 Hz, H-1); 2.43 (2H, dt, *J* = 6.6 y 1.8 Hz, H-1″); 2.17 (2H, m, H-4); 1.92 (1H, m, H-5); 1.68 (3H, s, 3-Me); 1.55 (2H, q, *J* = 7.4 Hz; H-2″); 1.43 (3H, s, H-8); 1.10 (3H, s, H-9); 0.88 (3H, t, *J* = 6.7 Hz, H-5″).

^13^C RMN (100 Mz, CDCl_3_, δ, ppm): 154.61 (C-2ʹ); 154.20 (C-6ʹ); 142.73 (C-4ʹ); 134.19 (C-3); 123.76 (C-2); 109.93 (C-1ʹ); 109.04 (C-5ʹ); 107.59 (C-3ʹ); 77.25 (C-7); 45.75 (C-6); 35.42 (C-1″); 33.54 (C-1); 31.47 (C-3″); 31.14 (C-4); 30.60 (C-2″); 27.50 (C-8); 24.96 (C-5); 23.32 (C-3-Me); 22.49 (C-4″); 19.20 (C-9); 13.97 (C-5″).

##### Cannabinol CBN

^1^H RMN (400 Mz, CDCl_3_, δ, ppm): 8.18 (1H, sa, H-2); 7.14 (1H, d, *J* = 7.9 Hz, H-5); 7.08 (1H, dd, *J* = 7.8 y 1.0 Hz, H-4); 6.43 (1H, d, *J* = 1.4 Hz, H-5ʹ), 6.29 (1H, d, *J* = 1.4 Hz, H-3ʹ); 6.25 (1H, s, 2ʹ-OH); 2.38 (3H, s, 3-Me); 1.60 (6H, s, H-8 y H-9), 1.32 (6H, m, H-3″ y H-4″); 0.86 (3H, t, *J* = 6.5 Hz, H-5″).

^13^C RMN (100 Mz, CDCl_3_, δ, ppm): 154.79 (C-2ʹ); 153.07 (C-6ʹ); 144.5 (C-4ʹ); 136.85 (C-3); 136.81 (C-6); 127.54 (C-4); 126.39 (C-2); 122.6 (C-5); 110.69 (C-5ʹ); 110.56 (C-1ʹ); 109.83 (C-3ʹ); 108.31 (C-1); 77.20 (C-7); 33.58 (C-1″); 31.44 (C-3″); 30.43 (C-2″); 27.07 (C-8 y C-9); 22.52 (C-4″); 21.50 (3-Me); 14.00 (C-5″).

##### 9-tetrahidrocannabinólic acid (THCA)

^1^H RMN (400 Mz, CDCl_3_, δ, ppm): 12.27 (1H, s, 2″-OH); 6.39 (1H, sa, H-2); 6.25 (1H, s, H-5ʹ); 3.23 (1H, m, H-1); 2.94 (1H, m, H-1″); 2.78 (1H, m, H-1″); 1.68 (3H, s, 3-Me); 1.44 (3H, s, H-8); 1.11 (3H, s, H-9); 0.90 (3H, t, J = 7.2 Hz, H-5″).

^13^C RMN (100 Mz, CDCl_3_, δ, ppm): 175.86 (CO_2_H); 164.66 (C-2ʹ); 159.69 (C-6ʹ); 146.85 (C-4ʹ); 133.83 (C-3), 123.59 (C-2); 112.57 (C-5ʹ); 109.82 (C-1ʹ); 102.27 (C-3’); 78.82 (C-7); 45.57 (C-6); 36.49 (C-1″); 33.43 (C-1); 32.00 (C-3″); 31.27 (C-2″), 31.19 (C-4); 27.36 (C-8); 24.97 (C-5); 23.32 (C-3-Me); 22.50 (C-4″); 19.49 (C-9); 14.05 (C-5″).

##### THC-Ac

^1^H RMN (400 Mz, CDCl_3_, δ, ppm): 6.55 (1H, d, J = 1.5 Hz, H-5ʹ); 6.40 (1H, d, J = 1.5 Hz, H-3ʹ); 5.97 (1H, sa, H-2); 3.05 (1H, d, J = 11.0 Hz, H-1); 2.48 (2H, t, J = 7.6 Hz, H-1″); 2.28 (3H, s, CO_2_CH_3_); 2.14 (2H, m, H-4); 1.66 (3H, s, 3-Me); 1.55 (2H, m, H-2″); 1.40 (3H, s, H-8); 1.09 (3H, s, H-9); 0.85 (3H, t, J = 5.4 Hz, H-5″).

##### CBN-Ac

^1^H RMN (400 Mz, CDCl_3_, δ, ppm): 7.81 (1H, sa, H-2); 7.14 (1H, d, *J* = 7.9 Hz, H-5); 7.08 (1H, dd,* J* = 7.9 Hz, H-4); 6.73 (1H, d, *J* = 1.3 Hz, H-5ʹ), 6.57 (1H, d, *J* = 1.3 Hz, H-3ʹ); 2.57 (2H, t, *J* = 7.6 Hz, H-1″); 2.37 (3H, s, 3-Me); 2.33 (3H, s, CO_2_CH_3_); 1.64 (3H, m, H-2″); 1.60 (6H, s, H-8 y H-9); 1.33 (6H, m, H-3″ y H-4″); 0.88 (3H, t, *J* = 6.8 Hz, H-5″).

^13^C RMN (100 Mz, CDCl_3_, δ, ppm): 169.03 (CO_2_CH_3_); 154.36 (C-2ʹ); 147.33 (C-6ʹ); 144.46 (C-4ʹ); 137.52 (C-6); 136.80 (C-3); 128.26 (C-2); 126.85 (C-1); 125.62 (C-4); 122.81 (C-5); 116.32 (C-5ʹ), 115.81 (C-3ʹ); 114.23 (C-1ʹ); 77.68 (C-7); 35.53 (C-1″); 31.42 (C-3″); 30.33 (C-2″); 26.92 (C-8 y C-9); 22.48 (C-4″); 21.47 (3-Me); 21.46 (CO_2_CH_3_); 13.99 (C-5″).

### Cell viability

**Fig. 3:**
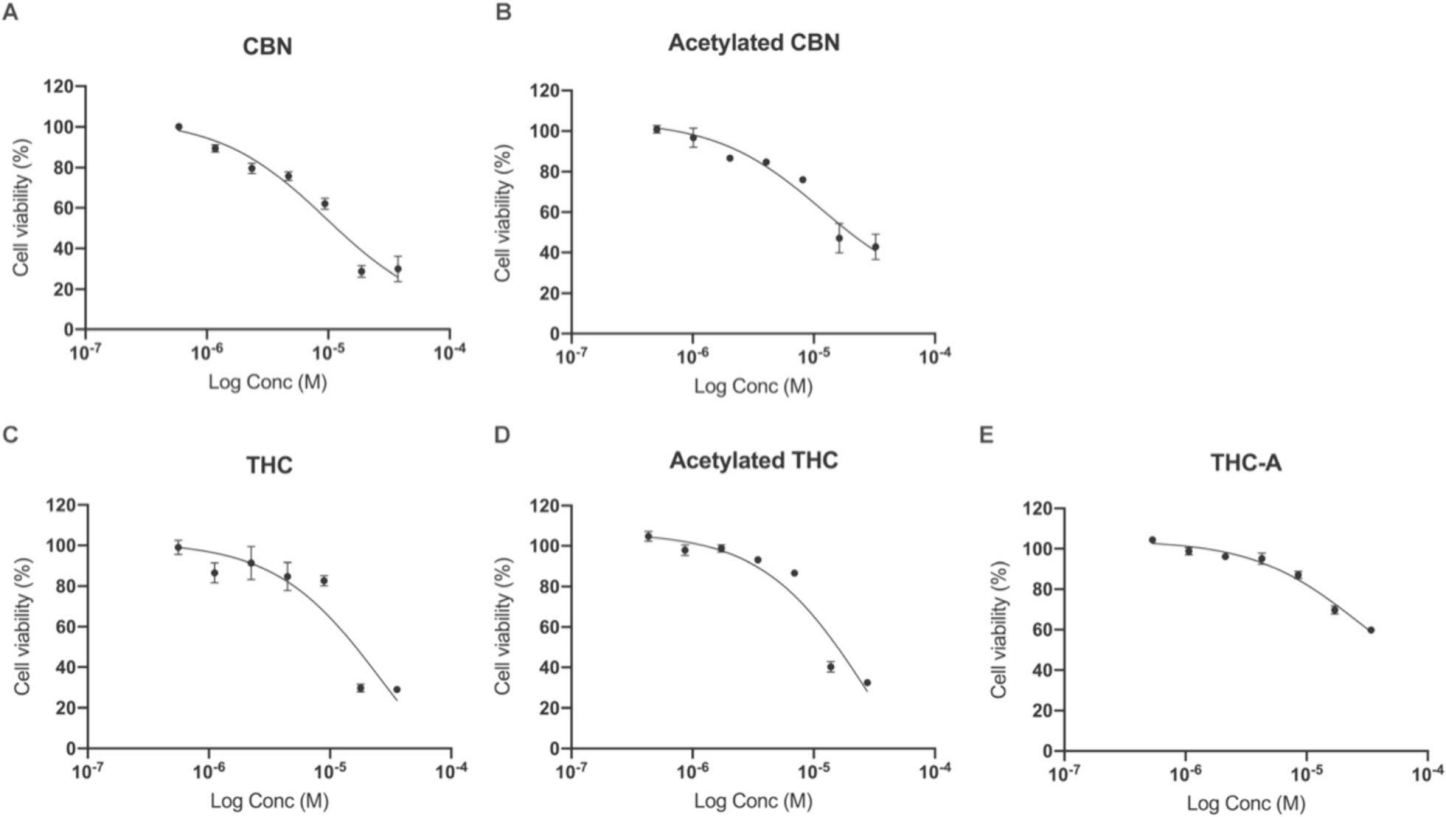
Cell viability of SHSY 5Y cells exposed to *C*BN (**A**), CBN-Ac (**B**), THC (**C**), THC-Ac (**D**) and THCA (**E**). Cells were exposed for 24 h to each condition and then the cell culture medium was replaced by resazurin 4 mg/L and the resulting fluorescence was read in a Varioskan multimode plate reader. The results represent three independent experiments, each one performed with triplicates (n = 3). Bars correspond to the standard error

The effect of hexane and acetyl acetate extracts, as well as phytocannabinoids and their derivatives were assessed on the neuroblastoma cell line, SHSY 5Y. After a 24 h exposition, to selected dilutions of the extracts and molecules, the resazurin assay was performed and the resulting viability calculated. The curves of viability for the phytocannabinoids and their acetylated counterparts are shown in Fig. 3. The viability curves for both extracts are presented as supplementary Fig. 1. The isolated compounds exhibited higher cytotoxicity than the original extracts, exhibiting THC and CBN the capacity to reduce cell viability to near 20%. Acetylation did not improve the cell killing capacity of the phytocannabinoids and THCA exhibited the lowest effect on the SHSY 5Y cells´ viability. The calculated IC_50_ for the compounds are presented in Table 2, this indicator reaffirms that acetylation did not improve the potency of the phytocannabinoids and give awareness of a higher activity of CBN over THC.

**Table 2:** IC50 for Phyto cannabinoids and their derivatives on the SHSY 5Y cells after 24 h exposure

Phyto cannabinoids	IC_50_ (µM)
CBN	9.5
CBN- Ac	11-6
THC	26.6
THC-Ac	28.4
THCA	26.6

### Caspases activity

**Fig. 4:**
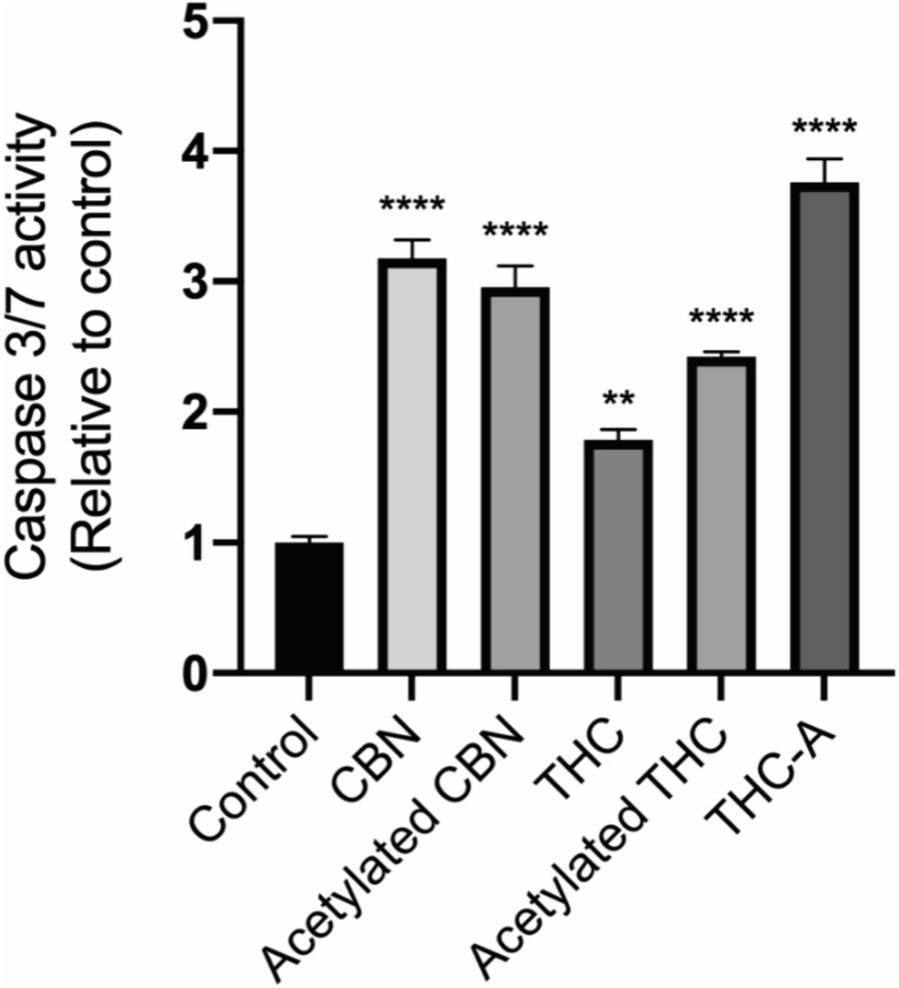
Activation of caspases 3 and 7 on the SH SY5Y cells exposed to phytocannabinoids. SH SY5Y CELL were exposed for 24 h to THC, THC-A, CBN, CBN-A and THCA or solvent control. Then the caspases 3 and 7 activity were assessed by the Promega GLO caspases kit, and luminescence was measured in a Varioskan multimode plate reader (** is p < 0.0026 and **** p < 0.0001). This experiment was performed once with triplicates for each assayed condition (n = 1)

To gain insight into the potential mechanism of the of the observed Phyto cannabinoids cytotoxicity caspases 3/7 activation was determined searching for evidence of an apoptotic mechanism of action. All the tested compounds exhibit the capacity to activate caspases 3 and 7, which was statistical significative compared with the control consisting in solvent-treated cells. The obtained results are presented in Fig. 4.

## Discussion

Natural products and their synthetic variations are still recognized as a principal source for anticancer drugs. Since 1981 to current times, almost 65% of the anticancer drugs are either natural or derived from natural molecules [14]. Phytocannabinoid, their hemisynthetic and synthetic derivatives have been largely studied to discover efficacious and selective compounds. In this research we aimed to examine the effects of *Cannabis sativa* L. extracts and isolated molecules, as well as their acetylated derivatives. We found that the Cannabis extracts, the phytocannabinoids and their acetylated versions inhibited the viability of the neuroblastoma cell line used as a model of study. However, the acetylated versions of THC and CBN, which were produced with the point to improve their potency, as reported previously for other receptor-mediated interactions, showed no more activity than their precursors when tested in vitro. Nevertheless, this modification could be potentially helpful if the molecule progresses towards in vivo assays, as acetylation of drugs would improve its lipophilicity which has been reported as a factor that increases permeability and thus absorption [15].

There was an overt cytotoxicity on the SHSY-5Y neuroblastoma for the evaluated extracts, isolated molecules and acetylated derivatives. We found IC_50_ values for the inhibition of SHSY 5Y cells' viability comparable with literature reports for other cell lines, thus for CBN we reported an IC_50_ of 9.5 µM for the SHSY 5Y cells whereas the literature has shown a range of 13–30 µM for the HuCCT1, IMR-5 and SK-N-AS cells and for THC we determined an IC_50_ value of 26.6 µM compared with 17,41 for HuCCT1 cells [5, 9]. This is the first report on the effect of acetylated derivatives of THC and CBN on a cancer cell line and therefore there is no data to contrast the results reported.

**Fig. 5:**
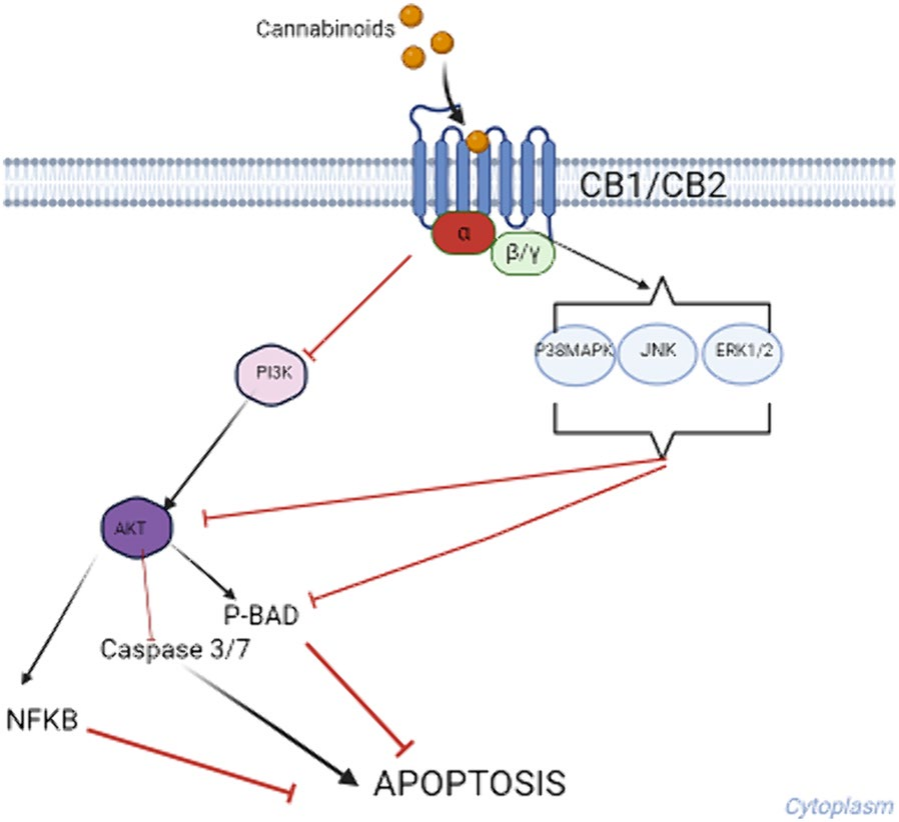
Potential signaling pathways for cannabinoids induction of apoptosis. The cannabinoids interact with protein G coupled CB1 or CB2 receptors. This activates MAP kinases which inhibit AKT and BAD phosphorylation. In parallel, PI3K is inhibited affecting AKT activation, BAD phosphorylation and NFKB activation, and releasing the inhibition of caspases 3 and 7 (drawed with Biorender on 30-03-2024 based on [9, 16, 17, 19]

These results are encouraging to advance with further studies of these compounds as potential anticancer molecules, including their mechanism of action. In this regard, the activation of caspases 3/7, two executioners caspases—activated by initiator and activated executioners caspases cleaving a linker that separate their small and large catalytic domains—are responsible for the typical morphology changes observed in apoptotic cells [16]. Since the assay we used is based on a common substrate for caspase 3 and 7, we cannot establish which one is involved. Nevertheless, because both act in tandem, thus caspase 3 activates caspase 7, and moreover in cells lacking caspase 3, it would be caspase 7, the first executioner caspase to be activated [17], our data supports the involvement of apoptosis as a partial mechanism of action. This agrees with literature reports showing that cannabinoids induce cell death by both apoptosis and autophagia [18]. CBN has attracted interest, recently showing to stimulate CB2 expression and thus inhibiting AKT activation [9], that mediates the suppression of proapoptotic molecules such as caspase 9 and the downstream caspase 3 [19]. Also, CBN has shown to increase Bax and reduce Bcl-2 expression and mitochondrial membrane potential, and cleavage of caspases 3 and 9 in a gastric cancer cell line, thereby causing apoptosis [9]. A model of a potential mechanism of action for phytocannabinoids is presented in Fig. 5. These observations are promissory since CBN is an abundant THC degradation product in the plant and it is weakly psychoactive.

Other perspective of the reported results could be that they suggest that consuming cannabis products recreationally could impose a potential danger for neuronal cells.

## Conclusion

Phyto cannabinoids exhibit higher viability inhibition capacity than the originating extracts. This activity is at least partially associated with an apoptosis inducing property. From all the tested molecules, CBN is a promising molecule for further studies as an anticancer agent for neuroblastoma treatment.

## Methods

### Raw vegetal material

Dr Rodrigo Díaz, holds resolution 5.544 given by the Chilean Public Health Institute the authorization to use *Cannabis sativa* L. for scientific purposes (Supplementary material, Fig Suppl. 11). The plant material utilized was obtained from a legal cultivar of Daya Foundation, a May 15th, 2018, harvest at Quinamávida, VII Región del Maule, Chile.

The plant material was dried and storage in closed containers under vacuum at room temperature and light and humidity protected.

### Extracts

50 g of grinded material were macerated in 1 L of either hexane or acetyl acetate for 72 h at room temperature. Next, they were filtered and concentrated at reduced pressure at 40 °C.

### Phytocannabinoids purification

A silica gel G-60 chromatography column was used as stationary phase and the mobile phase was a mixture hexane:acetyl acetate with increasing polarity over time.

### Extracts and Phyto cannabinoids characterization

#### High Performance Liquid Chromatography

For this analysis a previously validated method for cannabinoids detection was used (Tapia, 2017). Briefly, a HITACHI chromatograph ELITE LaChrom with an UV L2420 detector was used. The column was Kromasil 100-5-C8 (4.6 × 150 mm), the flow was 1.0 mL/min and the injection volumen was of 20 μL. The mobile phase was water: acetonitrile (20:80). The reading was made at 272 nm.

#### ^*1*^*H and *^*13*^*C Magnetic Nuclear Resonance*

A Bruker® Avance NEO 400 MHz spectrophotometer was used to perform NMR analysis to determine the chemical structures of THC, THCA, THC-Ac, CBN and CBN-Ac. The spectra were performed in a deuterated chloroform solution (CDCl_3_), using the residuals signals of CHCl_3_, δ = 7.26 ppm y δ = 77.0 ppm for ^1^H y ^13^C as references.

### Acetylated derivatives

THC or CBN dissolved in dichloromethane were mixed with acetic anhydride and dimethylaminopiridine and stirred for 1 h at room temperature. Next, a separation protocol was used to recover the organic phase to which anhydride magnesium sulfate was added and then filtered. This product was concentrated at reduced pressure and then purified by chromatography.

### Cell viability assay

The neuroblastoma human SH-SY5Y (94030304) cell line was purchased from the European Collection of Authenticated Cell Cultures (ECACC). They were kept in cell culture flasks in DMEM cell culture media supplemented with 10% fetal bovine sera, 10 µg/mL streptomycin, 100 µI/mL penicillin and 1% Glutamax at 37 °C in a Memmert incubator in a humidified 5% CO_2_ atmosphere.

For the viability the resazurin method was utilized based on the capability of viable cells to reduce it and give a fluorescent product, whose signal is proportional to the viable cells (O'Brien, 2000). For this, 5000 cells were exposed on a 96-well plate to serial dilutions of the extracts, THC, THCA, THC-Ac, CBN and CBN-Ac, and a vehicle control. Each condition was assayed in triplicates. After 24 h the cell culture media containing the different treatments was removed and replaced by cell culture media containing 4 mg/L resazurin. After 4 h, the fluorescence was read on a multimode plate reader Varioskan at 544 nm for excitation and 590 nm for emission. The percentage of viability was calculated with respect to the vehicle control. The IC_50_ was determined by lineal regression using the logarithm of the concentration versus the percentage of cell viability, using GraphPad Prism software.

### Caspases 3 y 7 activity

The Caspase-Glo® 3/7, Promega, kit was used following the manufacturer's directions. This assay was performed for THC, THCA, THC-Ac, CBN, CBN-Ac, the vehicle control and cells cultured on conventional cell culture media. The concentration of each molecule was twice their IC50. Briefly, 5000 SH-SY5Y cells were seeded in each well of a 96-wells opaque plate. After 24 h, the culture was exposed to the above-mentioned conditions for 24 h and then, the Caspase-GLO reagent was added, reading the luminescence in a multimode plate reader Varioskan flash.

### Statistical analysis

Data are presented using descriptive statistics as media ± SEM. In addition, the experimental groups were analyzed by one-way ANOVA followed by the comparative Dunnett´s test. Statistically significative differences were considered with a p < 0.05. The software GraphPad Prism was used for this analysis.

### Supplementary Information

**Supplementary material 1: Fig 1.** Cell viability of SHSY-5Y cells exposed to Acetate and Hexane extracts.Cells were exposed for 24 h to each condition and then the cell culture medium was replaced by resazurin 4mg/L and the resulting fluorescence was read in a Varioskan multimode plate reader. The results represent three independent experiments. Bars correspond to the standard error. **Fig 2.** 1H spectrum for THC. A Bruker® Avance NEO 400 MHz was used to register the spectrum. They were performed with deuterated chloroform using as reference the residual signals of CHCl3, δ=7,26 ppm and δ=77,0 ppm. **Fig 3.** 13C RMN spectrum for THC. A Bruker® Avance NEO 400 MHz was used to register the spectrum. They were performed with deuterated chloroform using as reference the residual signals of CHCl3, δ=7,26 ppm and δ=77,0 ppm. **Fig 4.** 1H spectrum for THC-Ac. A Bruker® Avance NEO 400 MHz was used to register the spectrum. They were performed with deuterated chloroform using as reference the residual signals of CHCl3, δ=7,26 ppm and δ=77,0 ppm. **Fig 5.** 1H RMN spectrum for THCA. A Bruker® Avance NEO 400 MHz was used to register the spectrum. They were performed with deuterated chloroform using as reference the residual signals of CHCl3, δ=7,26 ppm and δ=77,0 ppm. **Fig 6.** 13 C RMN spectrum for THCA. A Bruker® Avance NEO 400 MHz was used to register the spectrum. They were performed with deuterated chloroform using as reference the residual signals of CHCl3, δ=7,26 ppm and δ=77,0 ppm for 1H and 13C. **Fig 7.** 1H spectrum for CBN. A Bruker® Avance NEO 400 MHz was used to register the spectrum. They were performed with deuterated chloroform using as reference the residual signals of CHCl3, δ=7,26 ppm and δ=77,0 ppm. **Fig 8.** 13C spectrum for CBN. A Bruker® Avance NEO 400 MHz was used to register the spectrum. They were performed with deuterated chloroform using as reference the residual signals of CHCl3, δ=7,26 ppm and δ=77,0 ppm. **Fig 9.** 1H spectrum for CBN-Ac. A Bruker® Avance NEO 400 MHz was used to register the spectrum. They were performed with deuterated chloroform using as reference the residual signals of CHCl3, δ=7,26 ppm and δ=77,0 ppm. **Fig 10.** 13C spectrum for CBN-Ac. A Bruker® Avance NEO 400 MHz was used to register the spectrum. They were performed with deuterated chloroform using as reference the residual signals of CHCl3, δ=7,26 ppm and δ=77,0 ppm. **Fig 11.** Resolution for the use of Cannabis Sativa L.

## Data Availability

The authors declare that the data supporting the findings of this study are available within the paper and its Supplementary Information files. Should any raw data files be needed in another format they are available from the corresponding author upon reasonable request.

## Abbreviations

ANOVA: Analysis of variance
THC: Δ9-Tetrahidrocannabinol
CBD: Cannabidiol
CBN: Cannabinol
THCA: Acid form of Δ9-tetrahidrocannabinol
THC-Ac: Acetylated Δ9-tetrahidrocannabinol
CBN-Ac: Acetylated cannabinol
HPLC: High Performance Liquid Chromatography
IC50: 50% Inhibitory concentration
MNR: Magnetic Nuclear Resonance
