# Supplementary material for: Effect of Cannabis sativa L. extracts, phytocannabinoids and their acetylated derivates on the SHSY-5Y neuroblastoma cells’ viability and caspases 3/7 activation
Source: Biol Res. 2024 May 27;57:33. doi: 10.1186/s40659-024-00506-0 (PMC11129430; doi:10.1186/s40659-024-00506-0)
Supplement: Supplementary file 1 — Supplementary material 1: Fig 1. Cell viability of SHSY-5Y cells exposed to Acetate and Hexane extracts.Cells were exposed for 24 h to each condition and then the cell culture medium was replaced by resazurin 4mg/L and the resulting fluorescence was read in a Varioskan multimode plate reader. The results represent three independent experiments. Bars correspond to the standard error. Fig 2. 1H spectrum for THC. A Bruker® Avance NEO 400 MHz was used to register the spectrum. They were performed with deuterated chloroform using as reference the residual signals of CHCl3, δ=7,26 ppm and δ=77,0 ppm. Fig 3. 13C RMN spectrum for THC. A Bruker® Avance NEO 400 MHz was used to register the spectrum. They were performed with deuterated chloroform using as reference the residual signals of CHCl3, δ=7,26 ppm and δ=77,0 ppm. Fig 4. 1H spectrum for THC-Ac. A Bruker® Avance NEO 400 MHz was used to register the spectrum. They were performed with deuterated chloroform using as reference the residual signals of CHCl3, δ=7,26 ppm and δ=77,0 ppm. Fig 5. 1H RMN spectrum for THCA. A Bruker® Avance NEO 400 MHz was used to register the spectrum. They were performed with deuterated chloroform using as reference the residual signals of CHCl3, δ=7,26 ppm and δ=77,0 ppm. Fig 6. 13 C RMN spectrum for THCA. A Bruker® Avance NEO 400 MHz was used to register the spectrum. They were performed with deuterated chloroform using as reference the residual signals of CHCl3, δ=7,26 ppm and δ=77,0 ppm for 1H and 13C. Fig 7. 1H spectrum for CBN. A Bruker® Avance NEO 400 MHz was used to register the spectrum. They were performed with deuterated chloroform using as reference the residual signals of CHCl3, δ=7,26 ppm and δ=77,0 ppm. Fig 8. 13C spectrum for CBN. A Bruker® Avance NEO 400 MHz was used to register the spectrum. They were performed with deuterated chloroform using as reference the residual signals of CHCl3, δ=7,26 ppm and δ=77,0 ppm. Fig 9. 1H spectrum for CBN-Ac. A Bruker® Avance NEO 400 MHz was us [file 40659_2024_506_MOESM1_ESM.pdf]

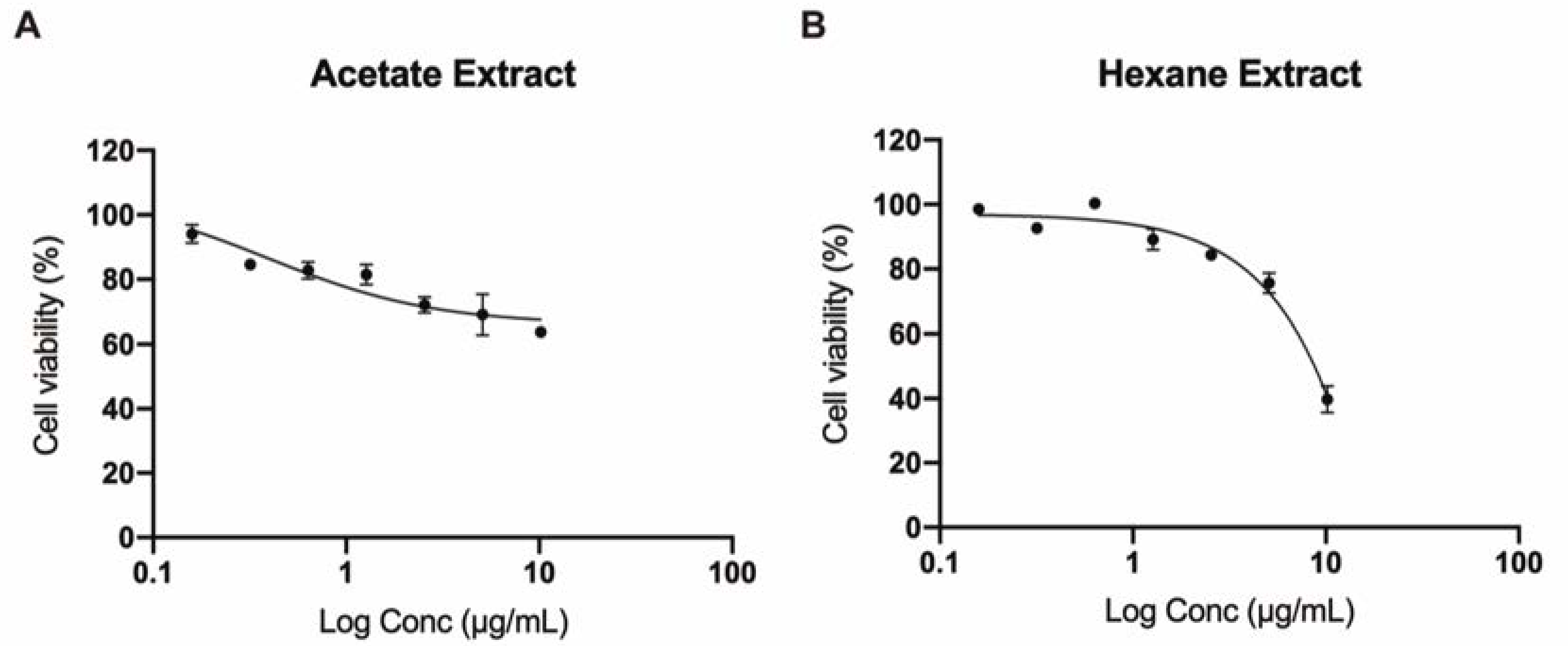
A
Acetate Extract
120
100
80
60
40
20
0
Cell viability (%)
0.1
1
10
100
Log Conc (µg/mL)
B
Hexane Extract
120
100
80
60
40
20
0
Cell viability (%)
0.1
1
10
100
Log Conc (µg/mL)

MCO 16
CDCl3

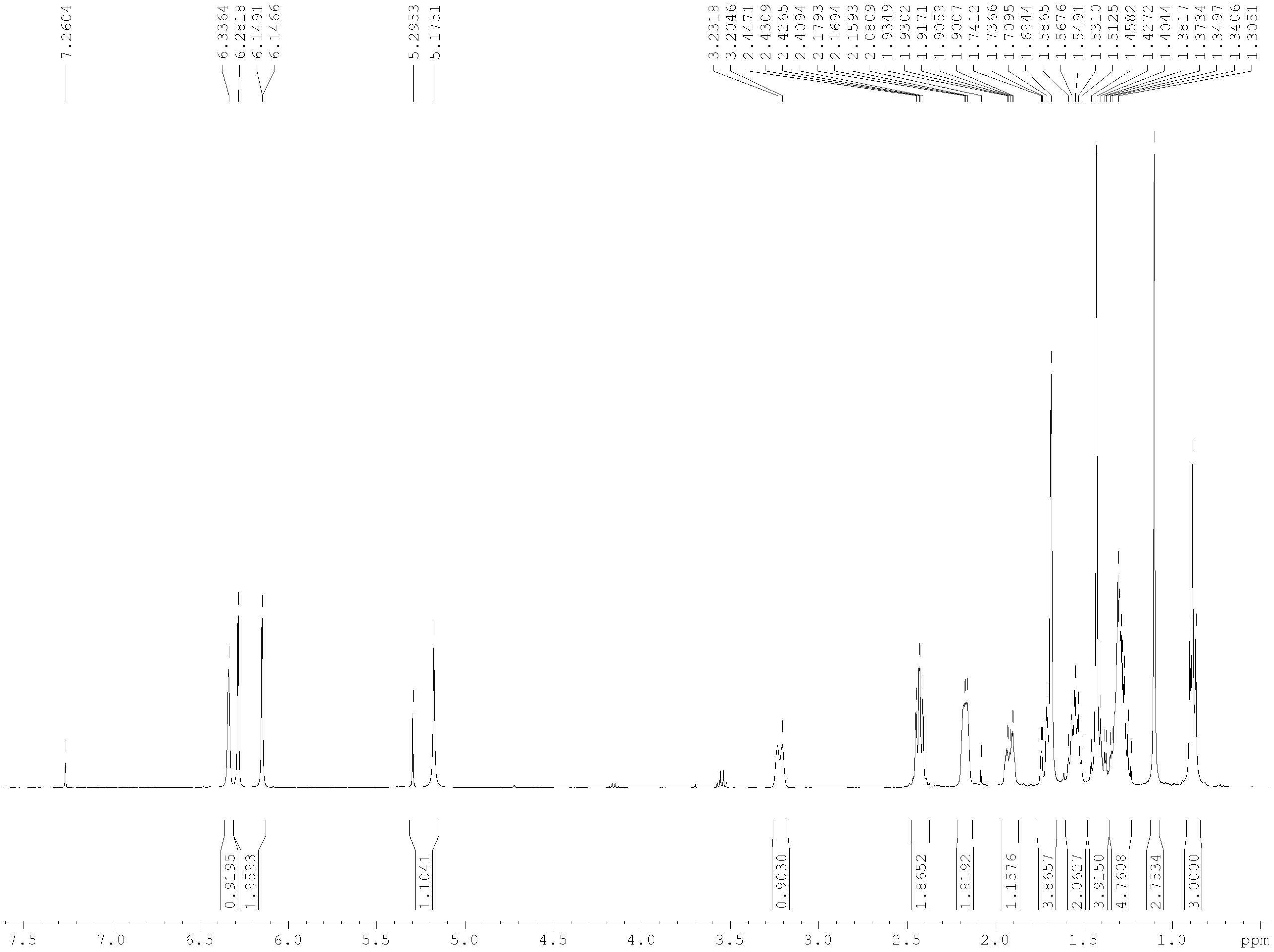

Current Data Parameters
NAME Mauricio Cuellar
EXPNO 28
PROCNO 1

F2 - Acquisition Parameters
Date_ 20210826
Time 14.32 h
INSTRUM Avance
PROBHD Z8202_0253 (BB
PULPROG zg30
TD 32768
SOLVENT CDCl3
NS 8
DS 2
SWH 4854.369 Hz
FIDRES 0.296287 Hz
AQ 3.3751040 sec
RG 30.7447
DW 103.000 usec
DE 11.68 usec
TE 0 K
D1 1.00000000 sec
TD0 1
SFO1 400.1442408 MHz
NUC1 1H
P0 2.33 usec
P1 7.00 usec
PLW1 15.52000046 W

F2 - Processing parameters
SI 65536
SF 400.1420097 MHz
WDW EM
SSB 0
LB 0.30 Hz
GB 0
PC 1.00

MCO 16
CDCl3

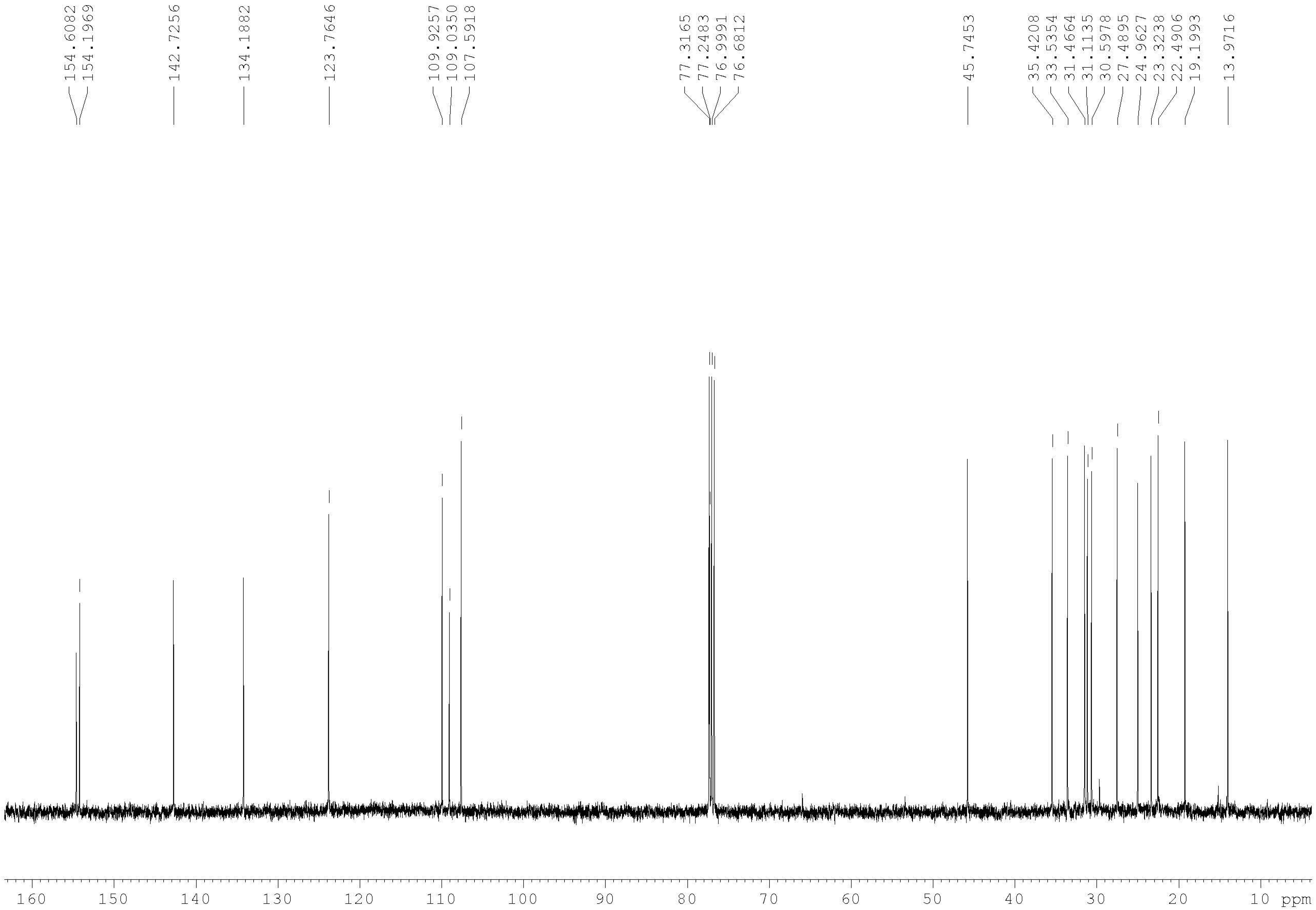

Current Data Parameters

NAME	Mauricio Cuellar
EXPNO	29
PROCNO	1

F2 - Acquisition Parameters

Date_	20210826
Time	14.43 h
INSTRUM	Avance
PROBHD	Z8202_0253 (BB
PULPROG	zgpg30
TD	65536
SOLVENT	CDCl3
NS	256
DS	4
SWH	23809.523 Hz
FIDRES	0.726609 Hz
AQ	1.3762560 sec
RG	3.25
DW	21.000 usec
DE	6.50 usec
TE	0 K
D1	1.00000000 sec
D11	0.03000000 sec
TD0	1
SFO1	100.6258475 MHz
NUC1	13C
P0	5.00 usec
P1	15.00 usec
PLW1	100.09999847 W
SFO2	400.1436006 MHz
NUC2	1H
CPDPRG[2	waltz65
PCPD2	90.00 usec
PLW2	15.52000046 W
PLW12	0.09388900 W
PLW13	0.04722500 W

F2 - Processing parameters

SI	32768
SF	100.6157948 MHz
WDW	EM
SSB	0
LB	1.00 Hz
GB	0
PC	1.40

THCA
CDCl3

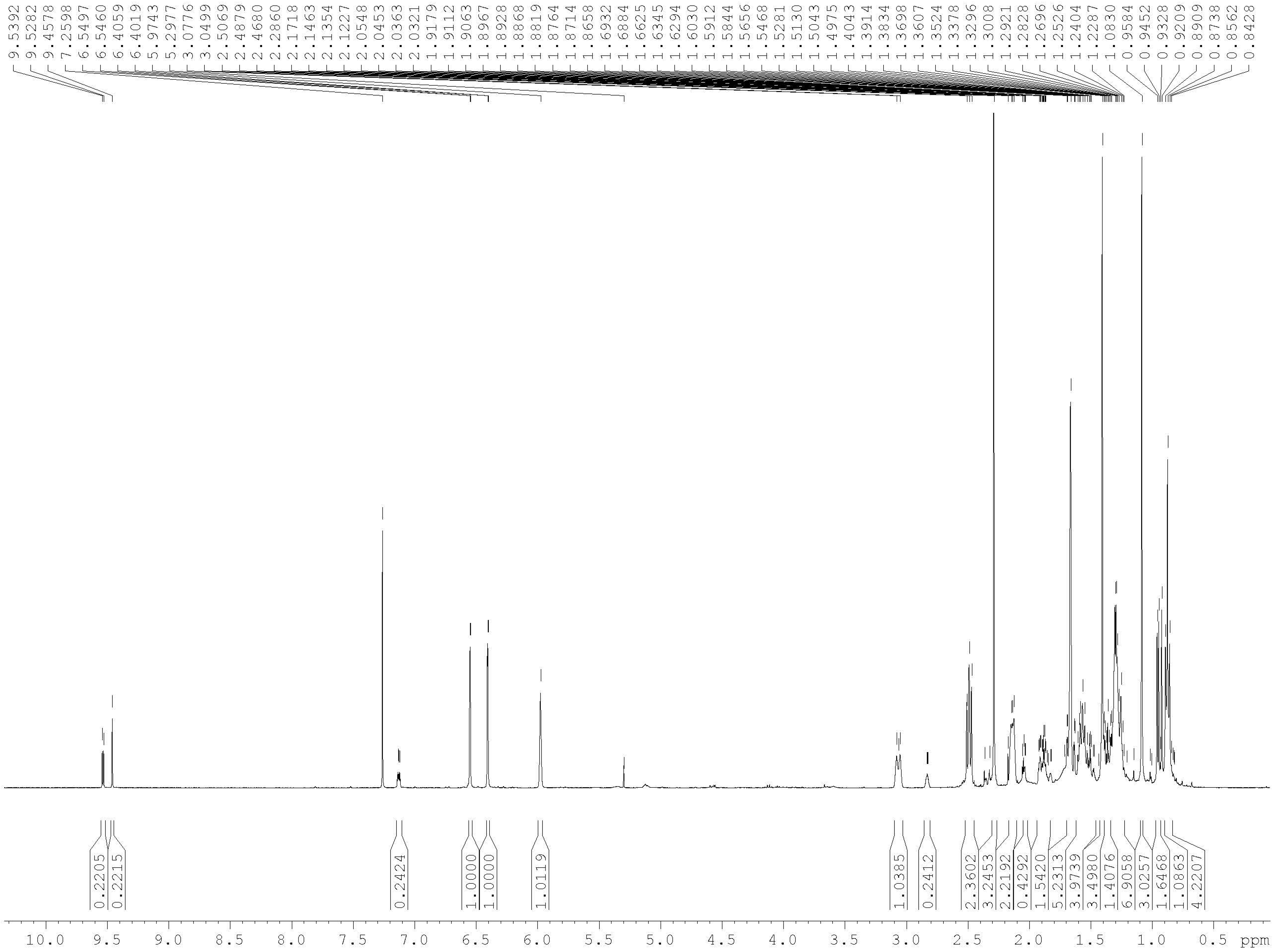

Current Data Parameters

NAME	Mauricio Cuellar
EXPNO	446
PROCNO	1

F2 - Acquisition Parameters

Date_	20220819
Time	8.33 h
INSTRUM	Avance
PROBHD	Z8202_0253 (BB
PULPROG	zg30
TD	32768
SOLVENT	CDCl3
NS	8
DS	2
SWH	4854.369 Hz
FIDRES	0.296287 Hz
AQ	3.3751040 sec
RG	101
DW	103.000 usec
DE	11.68 usec
TE	0 K
D1	1.00000000 sec
TD0	1
SFO1	400.1442408 MHz
NUC1	1H
P0	2.33 usec
P1	7.00 usec
PLW1	15.52000046 W

F2 - Processing parameters

SI	65536
SF	400.1420100 MHz
WDW	EM
SSB	0
LB	0.30 Hz
GB	0
PC	1.00

T-2
CDCl3

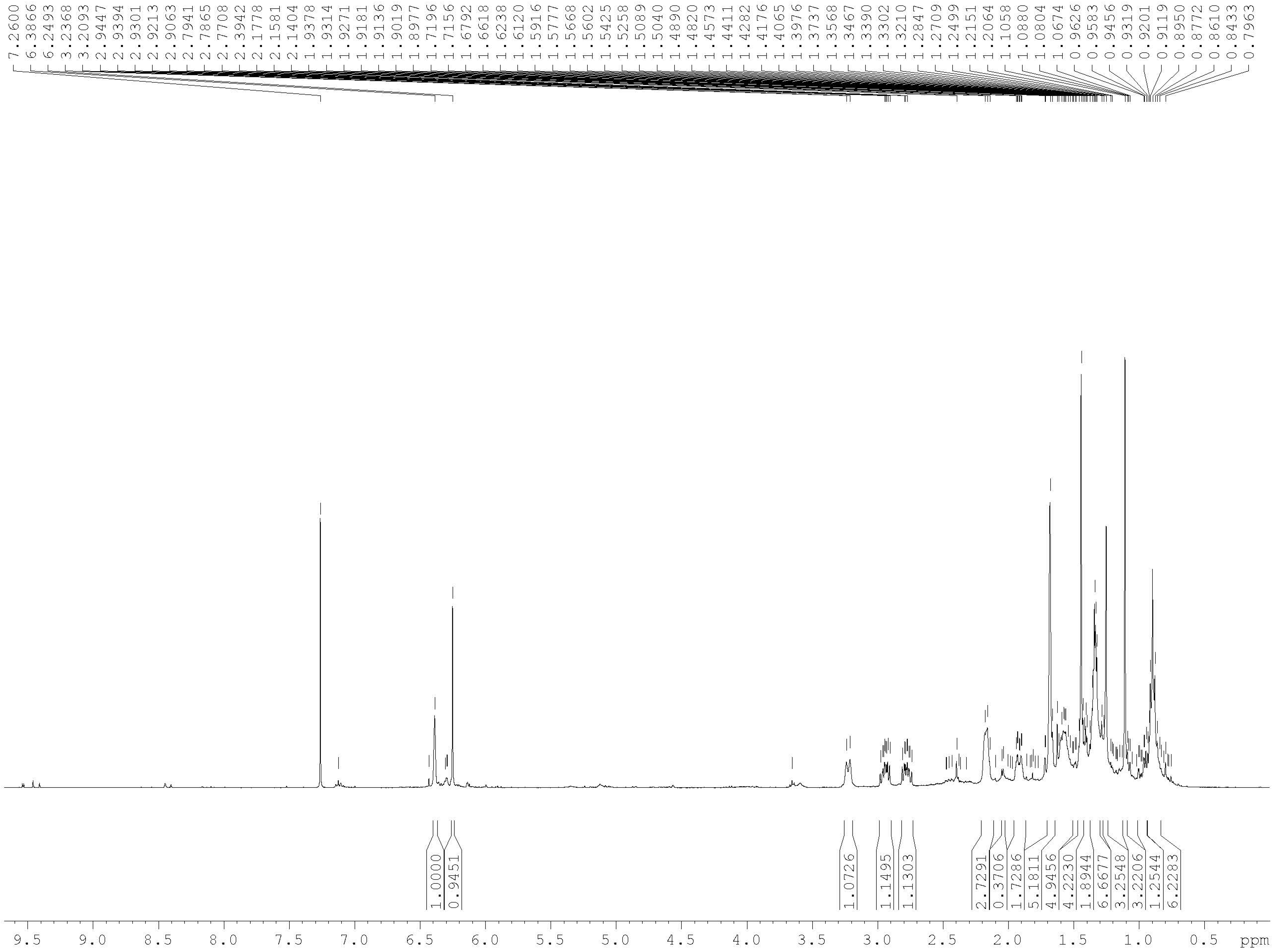

Current Data Parameters

NAME	Mauricio Cuellar
EXPNO	510
PROCNO	1

F2 - Acquisition Parameters

Date_	20221107
Time	14.44 h
INSTRUM	Avance
PROBHD	Z8202_0253 (BB
PULPROG	zg30
TD	32768
SOLVENT	CDCl3
NS	8
DS	2
SWH	4854.369 Hz
FIDRES	0.296287 Hz
AQ	3.3751040 sec
RG	101
DW	103.000 usec
DE	11.68 usec
TE	0 K
D1	1.00000000 sec
TD0	1
SFO1	400.1442408 MHz
NUC1	1H
P0	2.33 usec
P1	7.00 usec
PLW1	15.52000046 W

F2 - Processing parameters

SI	65536
SF	400.1420097 MHz
WDW	EM
SSB	0
LB	0.30 Hz
GB	0
PC	1.00

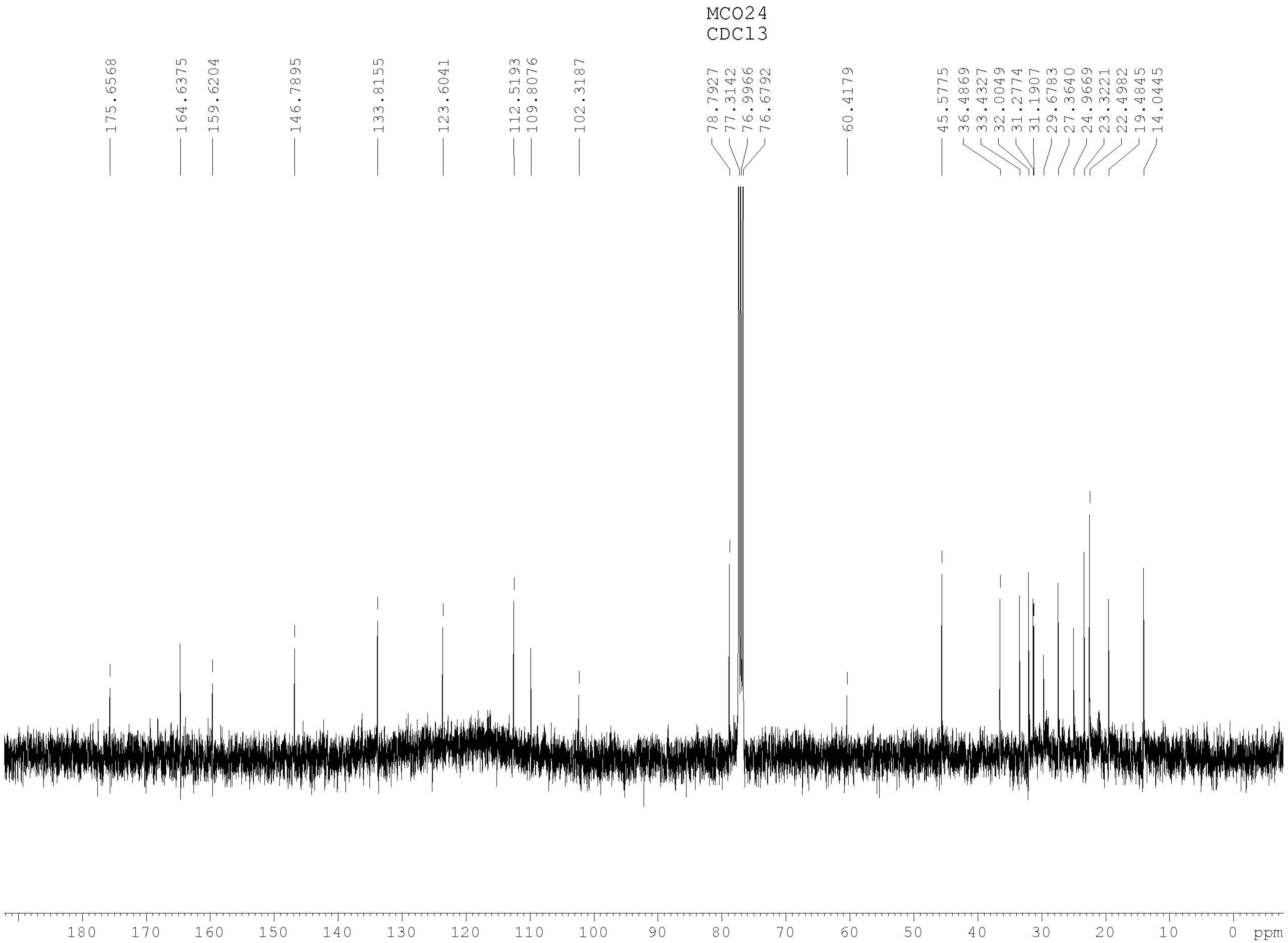

Current Data Parameters
NAME Mauricio Cuellar
EXPNO 78
PROCNO 1

F2 - Acquisition Parameters
Date_ 20211006
Time 23.46 h
INSTRUM Avance
PROBHD Z8202_0253 (BB
PULPROG zgpg30
TD 65536
SOLVENT CDCl3
NS 8000
DS 4
SWH 23809.523 Hz
FIDRES 0.726609 Hz
AQ 1.3762560 sec
RG 3.25
DW 21.000 usec
DE 6.50 usec
TE 0 K
D1 1.00000000 sec
D11 0.03000000 sec
TD0 1
SFO1 100.6258475 MHz
NUC1 13C
P0 5.00 usec
P1 15.00 usec
PLW1 100.09999847 W
SFO2 400.1436006 MHz
NUC2 1H
CPDPRG[2 waltz65
PCPD2 90.00 usec
PLW2 15.52000046 W
PLW12 0.09388900 W
PLW13 0.04722500 W

F2 - Processing parameters
SI 32768
SF 100.6157890 MHz
WDW EM
SSB 0
LB 1.00 Hz
GB 0
PC 1.40

C-1
CDCl3

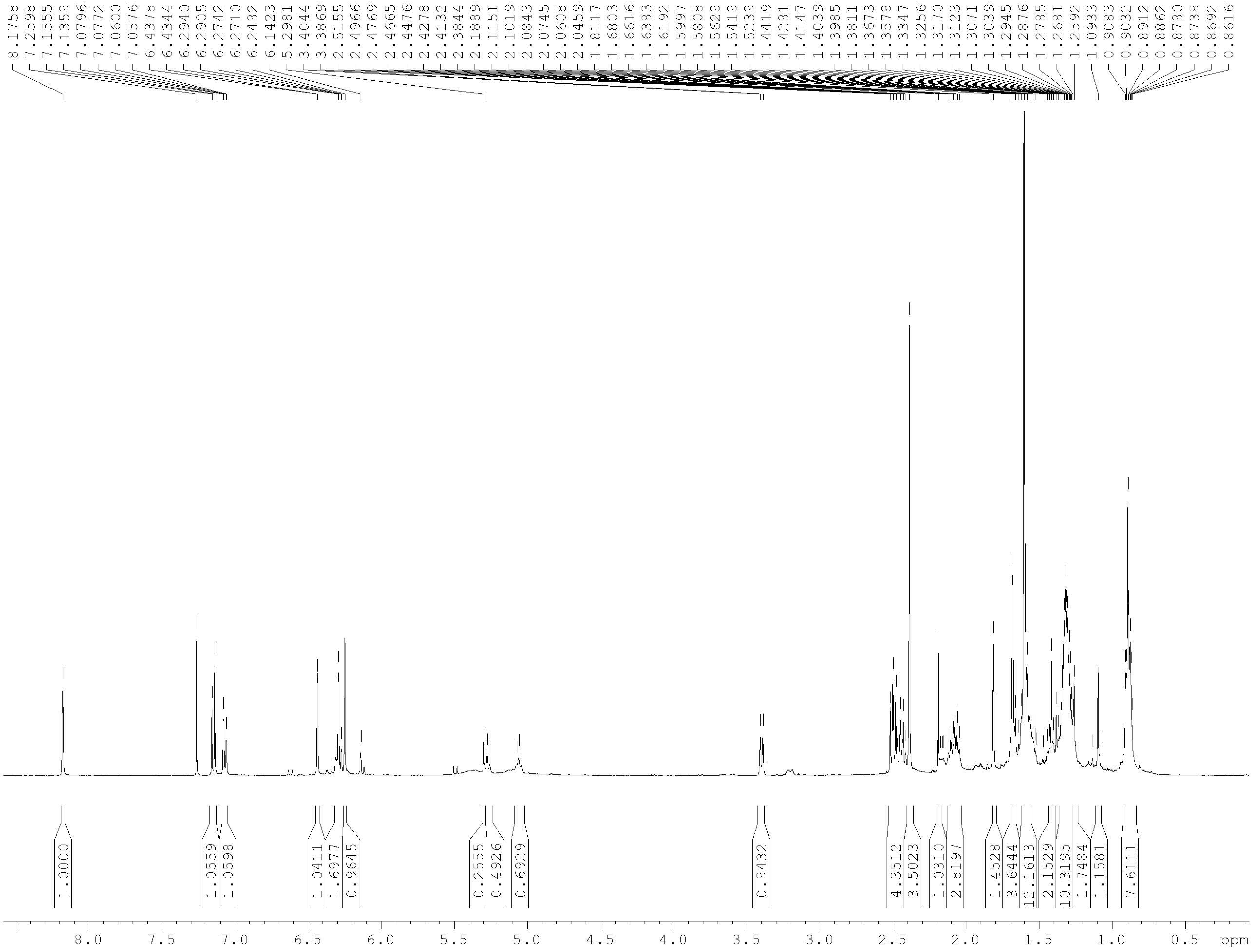

Current Data Parameters

NAME	Mauricio Cuellar
EXPNO	443
PROCNO	1

F2 - Acquisition Parameters

Date_	20220818
Time	11.52 h
INSTRUM	Avance
PROBHD	Z8202_0253 (BB
PULPROG	zg30
TD	32768
SOLVENT	CDCl3
NS	8
DS	2
SWH	4854.369 Hz
FIDRES	0.296287 Hz
AQ	3.3751040 sec
RG	90.5
DW	103.000 usec
DE	11.68 usec
TE	0 K
D1	1.00000000 sec
TD0	1
SFO1	400.1442408 MHz
NUC1	1H
P0	2.33 usec
P1	7.00 usec
PLW1	15.52000046 W

F2 - Processing parameters

SI	65536
SF	400.1420099 MHz
WDW	EM
SSB	0
LB	0.30 Hz
GB	0
PC	1.00

C-1
CDCl3

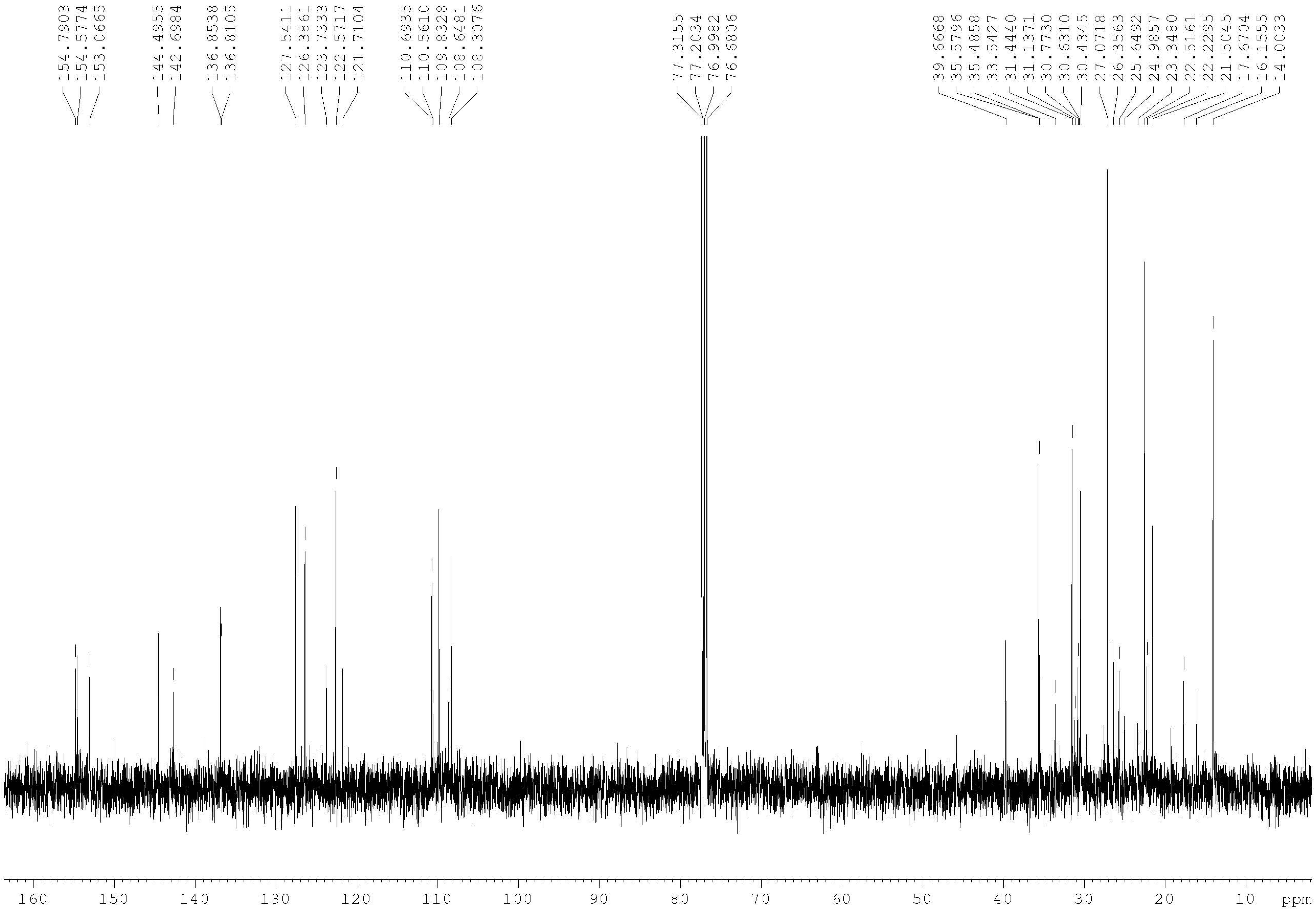

Current Data Parameters
NAME Mauricio Cuellar
EXPNO 449
PROCNO 1

F2 - Acquisition Parameters
Date_ 20220819
Time 10.53 h
INSTRUM Avance
PROBHD Z8202_0253 (BB
PULPROG zgpg30
TD 65536
SOLVENT CDCl3
NS 512
DS 4
SWH 23809.523 Hz
FIDRES 0.726609 Hz
AQ 1.3762560 sec
RG 3.2
DW 21.000 usec
DE 6.50 usec
TE 0 K
D1 1.00000000 sec
D11 0.03000000 sec
TD0 1
SFO1 100.6258475 MHz
NUC1 13C
P0 5.00 usec
P1 15.00 usec
PLW1 100.09999847 W
SFO2 400.1436006 MHz
NUC2 1H
CPDPRG[2 waltz65
PCPD2 90.00 usec
PLW2 15.52000046 W
PLW12 0.09388600 W
PLW13 0.04722400 W

F2 - Processing parameters
SI 32768
SF 100.6157903 MHz
WDW EM
SSB 0
LB 1.00 Hz
GB 0
PC 1.40

C-A
CDCl3

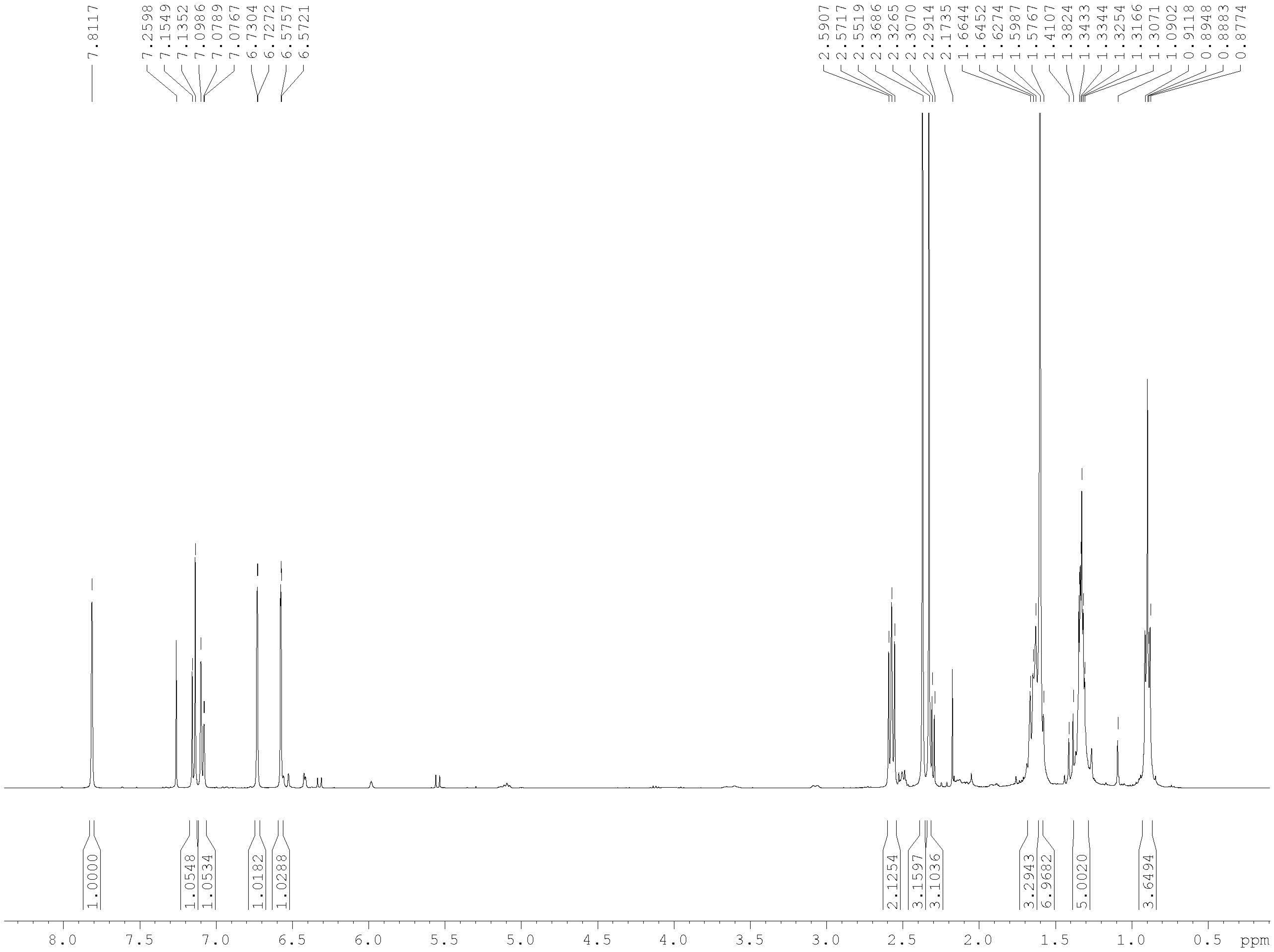

Current Data Parameters
NAME Mauricio Cuellar
EXPNO 457
PROCNO 1

F2 - Acquisition Parameters
Date_ 20220824
Time 15.42 h
INSTRUM Avance
PROBHD Z8202_0253 (BB
PULPROG zg30
TD 32768
SOLVENT CDCl3
NS 8
DS 2
SWH 4854.369 Hz
FIDRES 0.296287 Hz
AQ 3.3751040 sec
RG 71.8
DW 103.000 usec
DE 11.68 usec
TE 0 K
D1 1.00000000 sec
TD0 1
SFO1 400.1442408 MHz
NUC1 1H
P0 2.33 usec
P1 7.00 usec
PLW1 15.52000046 W

F2 - Processing parameters
SI 65536
SF 400.1420100 MHz
WDW EM
SSB 0
LB 0.30 Hz
GB 0
PC 1.00

TA-B
CDCl3

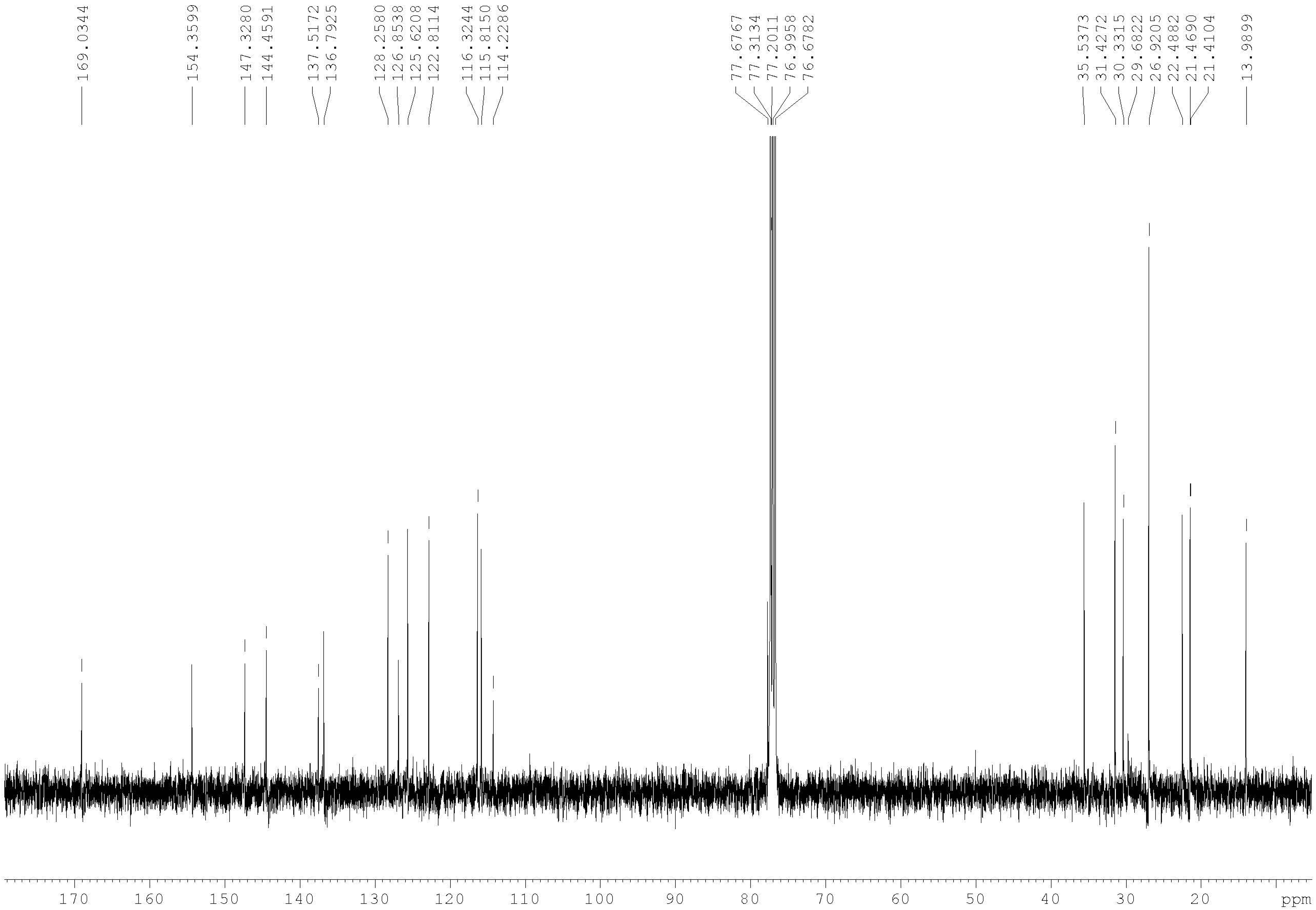


```
Current Data Parameters
NAME     Mauricio Cuellar
EXPNO                 508
PROCNO                  1

F2 - Acquisition Parameters
Date_            20221103
Time                21.32 h
INSTRUM            Avance
PROBHD   Z8202_0253 (BB
PULPROG           zgpg30
TD                 65536
SOLVENT            CDCl3
NS                  9024
DS                     4
SWH            23809.523 Hz
FIDRES          0.726609 Hz
AQ             1.3762560 sec
RG                   3.2
DW                21.000 usec
DE                  6.50 usec
TE                     0 K
D1            1.00000000 sec
D11           0.03000000 sec
TD0                    1
SFO1         100.6258475 MHz
NUC1                 13C
P0                  5.00 usec
P1                 15.00 usec
PLW1        100.09999847 W
SFO2         400.1436006 MHz
NUC2                  1H
CPDPRG[2         waltz65
PCPD2              90.00 usec
PLW2         15.52000046 W
PLW12         0.09388600 W
PLW13         0.04722400 W

F2 - Processing parameters
SI                 32768
SF           100.6157885 MHz
WDW                   EM
SSB                    0
LB                  1.00 Hz
GB                     0
PC                  1.40
```

Ref.: 12529/18
VBE/apa

5514 08.10.2018

SANTIAGO,

VISTO ESTOS ANTECEDENTES: la solicitud del Q. F. Héctor Rojas Campusano, Director Técnico de Knop Laboratorios S.A., por la que solicita autorización para trasladar desde Knop Laboratorios S.A., ubicado en Av. Industrial N° 1198, Quilpué, Región de Valparaíso, hasta la Universidad de Valparaíso (QUIFAC), ubicada en Av. Gran Bretaña N° 1093, Playa Ancha, Valparaíso, la muestra de ***Hierba Cannabis Sativa*** indicada en la parte resolutiva; y

CONSIDERANDO: que la muestra será trasladada a la Universidad de Valparaíso (QUIFAC), para proyecto de investigación; y

TENIENDO PRESENTE: lo dispuesto en el Código Sanitario, Decreto con Fuerza de Ley N° 725 de 1968; los Reglamentos de Estupefacientes y de Productos Psicotrópicos, aprobados por los Decretos Supremos N° 404/83 y N° 405/83, del Ministerio de Salud; los artículos 59° letra b) y 61° letra b) del DFL N° 1 de 2005 y las facultades delegadas por la Resolución N° 977 de 19/03/2015 de la Dirección del Instituto de Salud Pública de Chile, dicto lo siguiente:

RESOLUCIÓN

1.-**AUTORÍZASE** a Knop Laboratorios S.A., para trasladar a la Universidad de Valparaíso (QUIFAC) la muestra de droga estupefaciente que a continuación se señala:

Droga a trasladar	Cantidad	Desde	Hasta
Hierba Cannabis Sativa	5230 gramos	Knop Laboratorios S.A. Av. Industrial N° 1198, Quilpué, Región de Valparaíso	Universidad de Valparaíso (QUIFAC) Av. Gran Bretaña N° 1093, Playa Ancha, Valparaíso

2.- **DÉJESE ESTABLECIDO** que el Q.F. Rodrigo Díaz V., Director Técnico de la Universidad de Valparaíso (QUIFAC), será responsable de la tenencia y almacenamiento del producto en las nuevas dependencias.

ANÓTESE Y COMUNÍQUESE

AGENCIA NACIONAL DE MEDICAMENTOS
Subdepartamento Control de Comercio Exterior, Estupefacientes y Psicotrópicos
INSTITUTO DE SALUD PÚBLICA DE CHILE

Q.F. CARLOS BRAVO GOLDSMITH
JEFE SUBDEPARTAMENTO CONTROL COMERCIO EXTERIOR,
ESTUPEFACIENTES Y PSICOTRÓPICOS
DEPARTAMENTO AGENCIA NACIONAL DE MEDICAMENTOS
INSTITUTO DE SALUD PÚBLICA DE CHILE

DISTRIBUCIÓN:
- Knop Laboratorios S.A.
- Universidad de Valparaíso (QUIFAC)
- Gestión de Trámites

INSTITUTO DE SALUD PÚBLICA DE CHILE
MINISTRO DE FE
Transcrito fielmente
Ministro de fe
